# Effect of psychotherapy on recurrence of events and quality of life in patients with vasovagal syncope

**DOI:** 10.1038/s41598-022-09513-1

**Published:** 2022-04-06

**Authors:** Renata Libanori Aleixo de Barros e Silva, Rubens Marcelo Volich, Pedro Gabriel Melo de Barros e Silva, Francisco Carlos da Costa Darrieux, Maurício Ibrahim Scanavacca, Denise Tessariol Hachul

**Affiliations:** 1 Instituto do Coração do Hospital das Clínicas da Faculdade de Medicina da Universidade de São Paulo (InCor HC-FMUSP), Av. Dr. Enéas Carvalho de Aguiar, 44-Cerqueira César, São Paulo, SP 05403-900 Brazil; 2 Instituto Sedes Sapientiae, São Paulo, Brazil; 3 Centro Universitário São Camilo, São Paulo, Brazil

**Keywords:** Psychology, Cardiology, Cardiovascular diseases, Neurological disorders, Psychiatric disorders, Quality of life

## Abstract

Emotional distress is related to recurrence of syncope compromising patient's quality of life (QoL). The aim of this study was to determine if weekly sessions of psychotherapy reduce recurrence of events and improve QoL by SF-36 among patients with refractory vasovagal syncope. A randomised controlled pilot trial including 10 patients with recurrent vasovagal syncope and positive tilt table test was conducted. Known cardiac disease and ongoing psychotherapeutic interventions were the main exclusion criteria. All patients received standard of care treatment. Additionally, after randomisation, half of the patients underwent weekly sessions of psychotherapy for 12 months. Analysis of recurrence of events and QoL showed no significant change in the control group but, in the intervention group, there was a significant reduction in the rate of near-syncope episodes per month (5.7 ± 1.4 × 1.7 ± 1.0; P < 0.01), syncope in 1 year (4.6 ± 0.9 × 1.0 ± 0.7; P < 0.01) and a significant improvement in the overall assessment of QoL (44.1 ± 10.0 vs. 70.3 ± 10.3, P < 0.01). In conclusion, patients with refractory vasovagal syncope undergoing regular psychotherapeutic intervention had less recurrence of events and improved their quality of life in 1 year.

**Trial Registration:** ClinicalTrials.gov number, NCT04252729.

## Introduction

Syncope is a common clinical condition, affecting approximately one third of the population in their lifetime^1–3^ and is related to approximately 3–5% of the hospital admissions in the emergency room^4–6^. Neurally mediated events are responsible for more than a half of the cases of syncope and predominantly affects young women^4–6^. The main cause of syncope is the classical vasovagal mechanism, a type of neurally mediated syncope in which there is a sudden collapse of the sympathetic autonomic nervous system activity and an increase in vagal tone, causing vasodilation with or without bradycardia^4–8^. This condition, despite being benign in terms of mortality, presents a relevant morbidity and the risk of recurrence is of approximately 30% in 3 years^4–6^. The number of previous episodes is the major predictor of recurrence and is also directly related to a poorer quality of life^9–11^. Thus, vasovagal syncope has an important impact on patients' quality of life (QoL), especially in refractory cases.

Different cardiovascular manifestations have been associated to neurological stimuli^4–8^. The central autonomic nervous system has reciprocal connections with insular cortex, limbic and cardiovascular systems. Vasovagal syncope (VVS), particularly, can be triggered by factors such as intense emotions, distressful situations, pain and fear^4–8^. In addition, the emotional status of the patient with refractory vasovagal syncope is often impaired^9–11^ which could be associated to a higher risk of new events, contributing to the perpetuation of symptoms.

General measures, such as increase in salt and water intake and lifestyle interventions like exercises, tilt training and counter pressure maneuvers are usually recommended in order to avoid recurrences^4–6^. Different drugs were tested, but their efficacy is limited^4–6^. Psychological approach is frequently mentioned as a possible intervention to improve symptoms, but there is a lack of randomised clinical trials evaluating the specific effect of regular psychotherapy sessions among patients with recurrent refractory VVS.

### Objectives

The current study aims to evaluate the effect of psychotherapy on quality of life and on the number of syncope and near-syncope events during 1 year of follow-up, in patients with recurrent vasovagal syncope refractory to standard recommended therapy.

## Methods

### Study design

This study was a randomised controlled pilot clinical trial, developed in the Heart Institute of Sao Paulo University (InCor-FMUSP), in São Paulo, Brazil. The study was funded by the Brazilian Federal Agency for Support and Evaluation of Graduate Education (CAPES) which supports academic projects of public interest and was approved by the institutional review board.

### Study participants

Patients were screened from the Outpatient Syncope Unit at the Heart Institute of São Paulo University, which is a public health care reference for the population of the state of Sao Paulo, Brazil. Among this group of patients, the participants were included if they met the following criteria:

#### Inclusion criteria

- Age ≥ 18 years old
- Diagnosis of syncope of vasovagal origin after confirmation of a compatible clinical history and a positive tilt table test (vasodepressor, cardioinhibitory or mixed reflex responses) with reproduction of the clinical symptoms;
- Recurrent episodes (at least two episodes in the previous 6 months) refractory to standard therapy;
- Absence of cardiac, neurological or systemic disease.

#### Exclusion criteria

- Severe and/or uncontrolled systemic comorbidity: Diabetes Mellitus, hypertension, amyloidosis, myasthenia, Parkinson’s Disease; Neurogenic Dysautonomia;
- Age < 18 years;
- Current psychotherapeutic follow-up;
- Cardiac or neurological diseases;
- Pregnancy.

### Study procedures

#### Patient selection

In a screening performed on a database of patients with recurrent syncope, after introduction of conventional treatment, 14 patients were invited to an in-person interview. Four patients did not accept to participate and ten patients fulfilled the inclusion criteria, signed the informed consent form, and were initially followed in order to collect the data regarding the rate of events pre-randomisation. This baseline information included the number of syncope events in the 12-month period pre-intervention (syncope registry) and the rate of near-syncope events per month in the average of 2 months before the randomisation (with weekly contacts to track these events).

#### Randomisation

**Figure 1:**
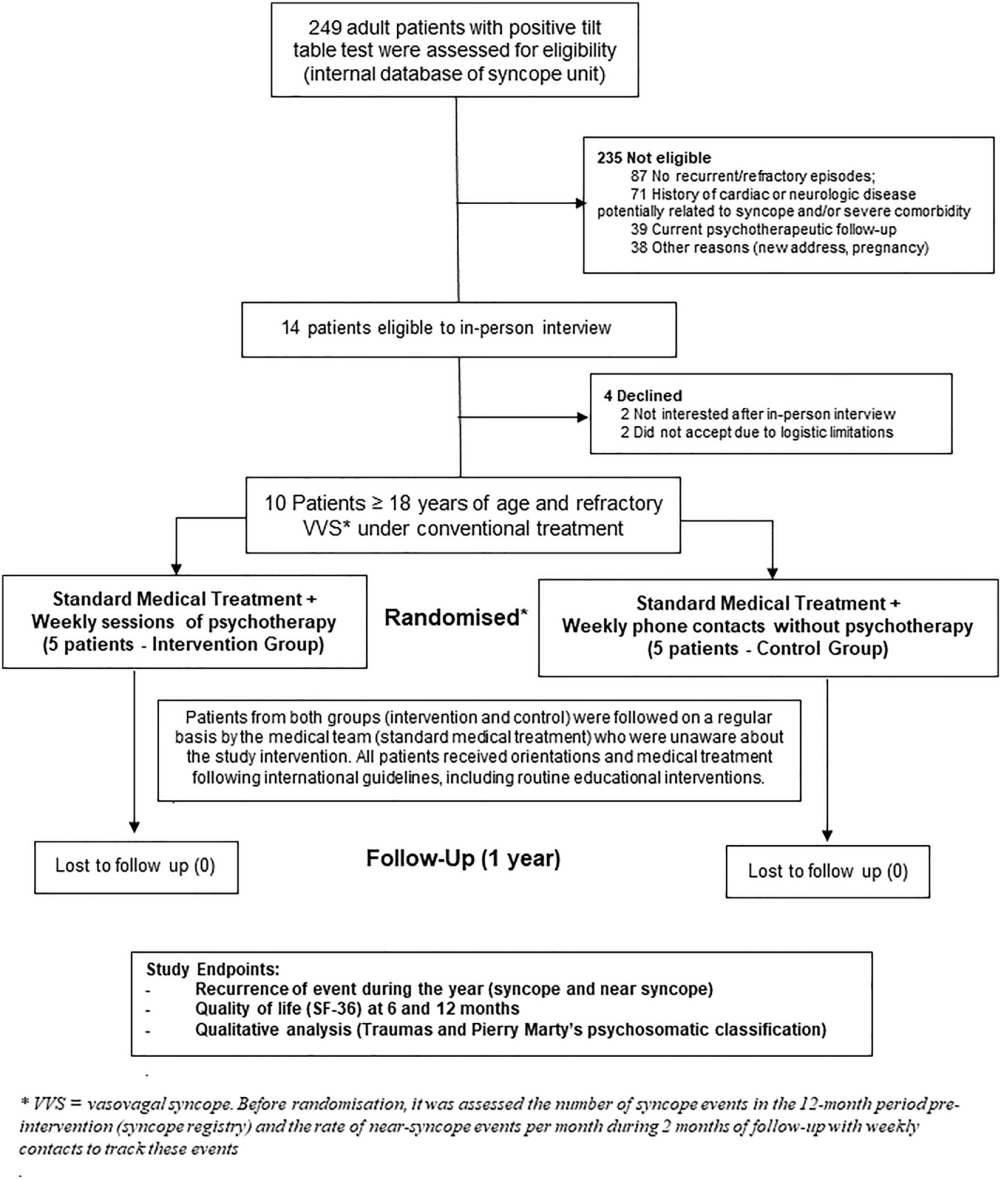
Recruitment, randomization and patient flow.

After the initial follow-up, eligible patients were randomised using a computerized method, without the possibility of predicting the group to which the patient would be allocated. Consequently, in a random and concealed way, half of the patients were selected for an open-label intervention (weekly sessions of psychotherapy), while the other half followed a conventional consultation model monitoring, without psychotherapy (Fig. 1).

#### Study procedures post-randomisation

The patients randomised to intervention were referred to weekly sessions of psychological assistance, all with the same psychologist. The sessions were performed both: in group sessions or individually, following a regular schedule of 42 sessions per patient in 1 year (from August 2017 to August 2018). No additional interventions (as educational or behavioural interventions) were performed during the sessions. The intervention was specifically psychotherapy following the theoretical line of Psychosomatic Psychoanalysis.

Patients from both groups (intervention and control) were followed on a regular basis by the same medical team who were unaware about the study intervention (Fig. 1). The medical team did not participate in the psychotherapy sessions. All patients (in both groups) received non-pharmacological interventions, including education, lifestyle modification, and reassurance regarding the benign nature of the condition during routine visits of medical care. Thus, all patients included in the study received a similar education therapy orientations and medical treatment following international guidelines^5,6^. These educational and pharmacological interventions were initiated in both groups before the inclusion. Both groups received weekly contact by the study team in order to collect information of potential events (syncope and near syncope). If the patient raised any question regarding the disease during these weekly contacts, a standard recommendation was performed in order to seek medical help with the same medical team. No patient had an implantable loop recorder.

### Study endpoints

#### Co-primary endpoints

- To evaluate the modification in the number of syncope and near-syncope events according to the participation in the weekly psychotherapeutic interventions or not, along 1 year.
- To evaluate the effect of psychotherapy on the overall quality of life of the patients, assessed by the SF-36 questionnaire, after 1 year of follow-up.

#### Secondary endpoints

- To evaluate the effect of psychotherapy on specific domains of quality of life assessed by the SF-36 questionnaire at 6 and 12 months in each group compared to baseline findings;
- To evaluate psychological traumatic events that occurred before the first syncope episodes.
- To evaluate the evolution of psychic structure during the psychotherapeutic sessions based on the Pierre Marty psychosomatic classification^12^.

### Data collection

Quantitative variables were collected at baseline, 6 months and 12 months, including demographic data, other medical problems, concomitant medications and quality of life. The analysis of SF-36 was performed following a standard script. All the clinical events were reported by the patients using text messages and confirmed during the weekly session in the intervention group, or by weekly phone calls in the control group.

### Sample size

There were no previous studies or evidence that could be used to estimate the effect of this intervention. An amount of ten patients with recurrent and refractory vasovagal syncope and severe clinical presentation were selected to test the hypothesis, as a pilot trial. The number of patients included would allow weekly sessions for the intervention group with the same psychologist and following the same approach, in order to avoid heterogeneity and to provide a more standardized therapy.

### Statistical analyses

Categorical variables were reported by the absolute and relative frequencies, and continuous variables were described by mean and standard deviation. Comparisons between groups in baseline characteristics of continuous and categorical variables were made using appropriate tests for each analysis (t test for continuous variables and Fisher's exact test for categorical variables). Regarding study outcomes, comparisons between groups and within groups were done by Brunner and Langer’s nonparametric tests for repeated measures^13,14^. All tests were two-tailed and, for the co-primary endpoints, P values < 0.025 were considered significant (rejection of the null hypothesis). All statistical analyses were performed using R software, version 4.0.2 (R Foundation for Statistical Computing).

### Qualitative analyses

The evaluation of phycological traumatic events preceding the first syncope episodes and the Pierre Marty’s psychosomatic classification^12^ were performed during the sessions by qualitative analysis. From the elaboration of narrations about trauma, patients perceive and understand traumatic events. To understand the evolution of the subjects' psychic structure, a psychosomatic investigation was carried out. The elements obtained from this investigation allow, according to Marty, to establish a psychosomatic classification by understanding the patient's psychic functioning according to a structural and economic perspective during psychotherapy.

### Ethical approval and informed consent

We declare that all methods were performed in accordance to international good clinical practices guidelines and national regulations. The project was submitted and approved by the Research Ethics Committee of the Hospital das Clinicas da Faculdade de Medicina da Universidade de São Paulo-HCFMUSP (CAAE: 57423816.6.0000.0068). All the patients signed free and informed consent forms before study procedures.

## Results

The groups were similar considering the baseline characteristics (Table 1). Overall, the study population was composed mainly by white (80%) female patients (70%), with the mean age of 47.4 (± 11.1) years-old and a tilt test showing a mixed mechanism of vasovagal reflex (80%). One patient was using calcium channel blocker due to hypertension and, regarding medications due to syncope, 40% were using fludrocortisone in both groups and none were using midodrine, which is a medication not available in Brazil (Table 1). No modification of medications was performed during the follow-up. All patients had prodromal symptoms and near-syncope episodes, in addition to recurrent syncope. Despite the prodromal symptoms, half of the patients had history of physical injuries related to syncope (Table 1). The overall adherence to the psychotherapeutic sessions was 97.6% (5 absences among the 210 attendance assessments in 1 year).

**Table 1:** Baseline characteristics of the patients.

Baseline characteristics	Intervention (n = 5)	Control (n = 5)	P value
Age, mean (± SD), years	48.8 (± 11.4)	46.0 (± 10.8)	0.70
**Race, No./total No. (%)**			
White	4/5 (80%)	4/5 (80%)	1.00
Mixed race	1/5 (20%)	1/5 (20%)	1.00
Female, No. /total No. (%)	4/5 (80%)	3/5 (60%)	1.00
Time from diagnosis, mean (± SD), months	25.4 (± 5.2)	26.0 (± 7.9)	0.89
**Type of vasovagal syncope, No. /total no. (%)**			
Mixed response	4/5 (80%)	4/5 (80%)	1.00
Cardioinhibitory	1/5 (20%)	0/5 (0%)	1.00
Vasodepressor	0/5 (0%)	1/5 (20%)	1.00
**Resting ECG, No. /total No. (%)**			
Normal, Sinus rhythm	5/5 (100%)	5/5 (100%)	1.00
Prodromes, No. /total No. (%)	5/5 (100%)	5/5 (100%)	1.00
Near-syncope, No. /total No. (%)	5/5 (100%)	5/5 (100%)	1.00
History of physical injuries related to syncope	3/5 (60%)	2/5 (40%)	1.00
**Comorbidities, No. /total No. (%)**			
Diabetes mellitus	2/5 (40%)	1/5 (20%)	1.00
Hypertension	0/5 (0%)	1/5 (20%)	1.00
Dyslipidemia	1/5 (20%)	1/5 (20%)	1.00
Hypothyroidism	1/5 (20%)	0/5 (0%)	1.00
**Medications, No. /total No. (%)**			
Fludrocortisone	2/5 (40%)	2/5 (40%)	1.00
Calcium channel blocker (diltiazem)	0/5 (0%)	1/5 (20%)	1.00

### Co-primary endpoints

#### Recurrence of syncope and near-syncope

**Figure 2:**
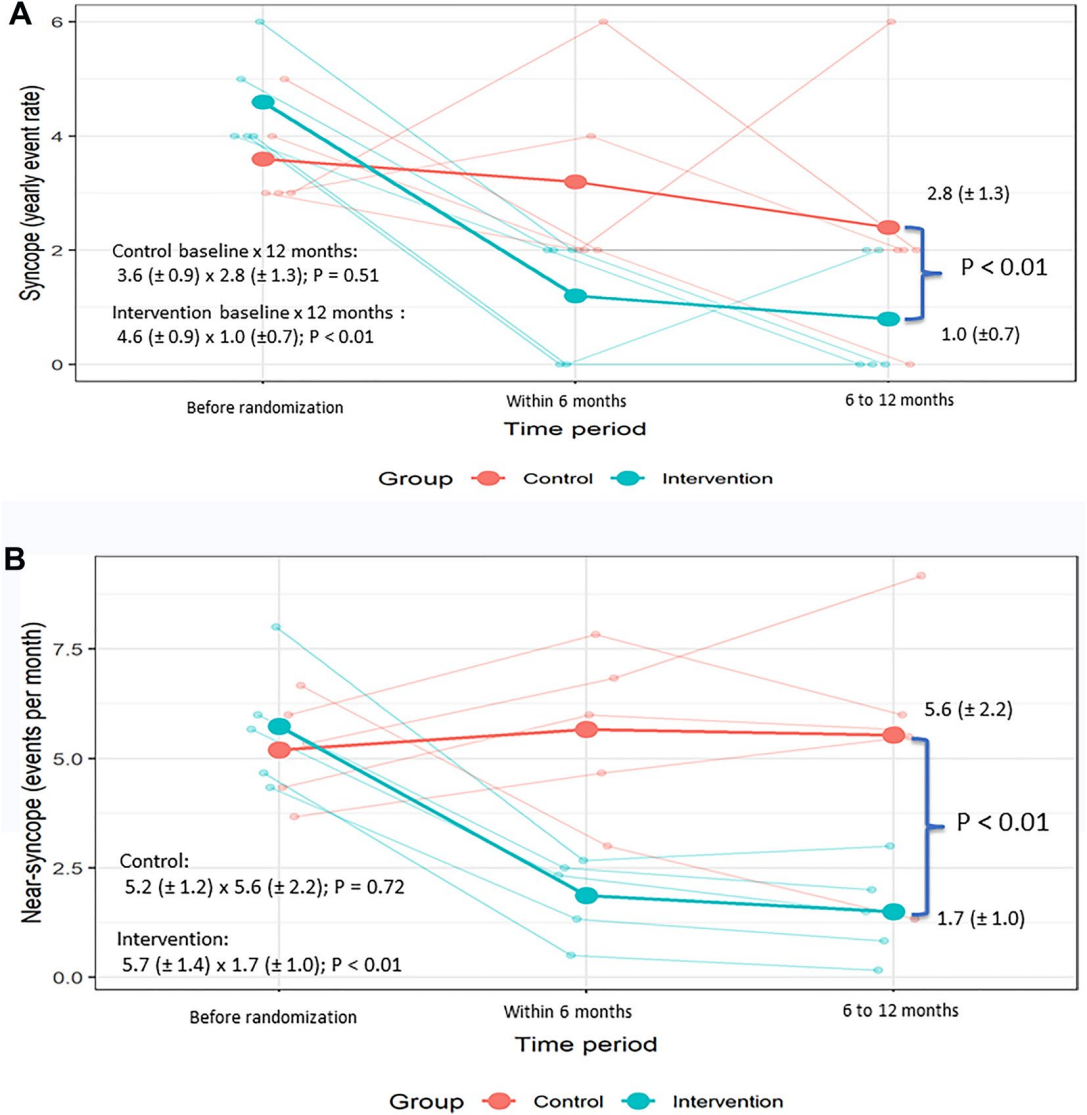
Comparison of event rate at baseline, 6 months and 12 months. (**A**) Syncope event rate (per year). (**B**) Near syncope event rate (per month). Each light line represents one patient while the dark (dense) line represents the average of the group (each group is identified by a different colour).

In 1 year, the recurrence of syncope and near-syncope events were different in both groups (Table 2; Fig. 2). In an individualized assessment, there was a numerical reduction in the frequency of episodes in all patients randomised to psychotherapeutic intervention, while in the control group only one patient improved symptoms in 1 year of follow-up (Fig. 2).

**Table 2:** Comparison between the average number of syncope and near-syncope prior to the randomisation versus during the year post-randomisation.

Therapeutic group	Before intervention	Year of intervention	P value
Average near-syncope per month	5.7 (± 1.4)	1.7 (± 1.0)	< 0.01
Average syncope per year	4.6 (± 0.9)	1.0 (± 0.7)	< 0.01

Control group	Before randomisation	Year of follow-up	P value
Average near syncope per month	5.2 (± 1.2)	5.6 (± 2.2)	0.72
Average syncopes per year	3.6 (± 0.9)	2.8 (± 1.3)	0.51

Values are means ± SDs.

Comparing the frequency before and after 1 year of follow-up, there was an average reduction, statistically significant, of 4.0 less near-syncope events per month (70% reduction) and 3.6 less syncope events per year in the intervention group (68.3% reduction), while in the control group, there was no difference in the average frequency of both events during follow-up (Table 2; Fig. 2). The improvement in the intervention group was achieved already in the first 6 months of psychotherapeutic sessions and continued in the last 6 months of intervention (Fig. 2).

Comparing both groups there was a statistically difference in 1 year of follow-up both in near syncope (5.6 ± 2.2 vs 1.7 ± 1.0; P < 0.01) and syncope (2.8 ± 1.3 vs 1 ± 0.7; P < 0.01) (Table 2; Fig. 2). Comparing the delta of change between the groups, the difference was also statistically significant between the groups both in near syncope (0.4 ± 2.8 vs − 4 ± 0.7; P = 0.02) and syncope (− 0.8 ± 2.2 vs − 3.6 ± 0.9; P < 0.01).

#### Effect of psychotherapy on the overall quality of life

**Figure 3:**
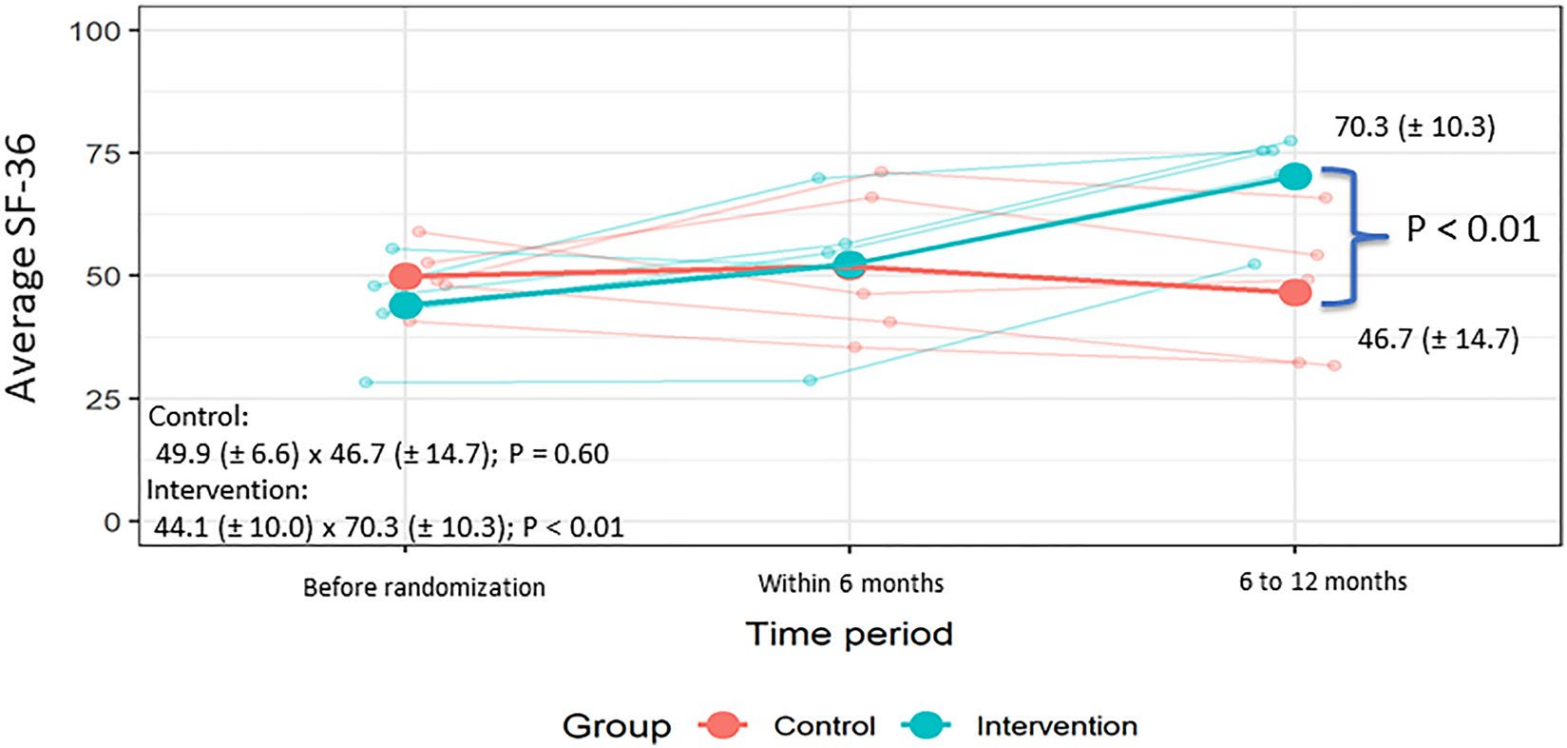
Comparison of average of SF-36 at baseline, 6 months and 12 months. Each light line represents one patient while the dark (dense) line represents the average of the group (each group is identified by a different colour).

In 1 year, there was no improvement in quality of life by SF-36 in the control group, but there was a statistically significant improvement in the psychotherapeutic group (Table 3; Fig. 3). Comparing the final results at 12 months between the 2 groups (Fig. 3), there was a statistically significant improvement in the patients that underwent psychotherapeutic intervention (46.7 ± 14.7 vs 70.3 ± 10.3; P < 0.01).

**Table 3:** Effect of psychotherapy on patient’s quality of life.

Therapeutic group	Baseline	1 year	P value
Physical functioning	68 ± 21.1	73 ± 19.9	< 0.01
Role-physical	40 ± 28.5	60 ± 28.5	< 0.01
Bodily pain	40.6 ± 18.2	70.6 ± 17.6	< 0.01
General health	44.8 ± 13.8	73 ± 11.4	< 0.01
Vitality	43 ± 5.7	59 ± 6.5	< 0.01
Social functioning	37.5 ± 12.5	62.5 ± 8.8	< 0.01
Role-emotional	26.7 ± 27.9	86.7 ± 18.3	< 0.01
Mental health	52 ± 16.2	77.6 ± 9.2	< 0.01
Average	44.1 ± 10.0	70.3 ± 10.3	< 0.01

Control group	Baseline	1 year	P value
Physical functioning	68 ± 13.5	63 ± 15.2	< 0.01
Role-physical	50 ± 17.7	35 ± 28.5	0.20
Bodily pain	51.6 ± 13.5	49.4 ± 8.8	0.81
General health	45.6 ± 19.6	46.6 ± 21	0.31
Vitality	46 ± 11.9	45 ± 13.7	0.85
Social functioning	52.5 ± 18.5	47.5 ± 16.3	0.54
Role-emotional	33.3 ± 23.6	33.3 ± 33.3	0.96
Mental health	52 ± 7.5	53.6 ± 10.8	0.63
Average	49.9 ± 6.6	46.7 ± 14.7	0.60

Values are means ± SDs.

### Secondary endpoints

In 1 year, there was no improvement in each of the 8 domains of SF-36 in the control group, but there was a statistically significant improvement identified in all domains in the psychotherapeutic group (Table 3). The improvement in SF-36 occurred already in the first 6 months of psychotherapeutic sessions and continued in the last 6 months of intervention (Fig. 3).

Regarding qualitative analysis, it was possible to identify traumatic events temporally related to the first syncope episodes in all patients that underwent psychotherapeutic sessions (Table 4). Using the Pierre Marty psychosomatic classification, it was possible to identify “poor mentalization” and “uncertain mentalization” that improved during the psychotherapeutic sessions (Table 5).

**Table 4:** Traumas preceding first syncope events detected during psychotherapeutic sessions.

	Traumas
Patient 1	Death of the father with whom he had a symbiotic relationship
Patient 2	Traumatic death of father in adolescence
Patient 3	Father's aggressiveness and mother's alienation
Patient 4	Death of the younger brother in childhood
Patient 5	Husband's sexual violence

**Table 5:** Pierre Marty’s nosological framework.

	Fundamental structures	Important habitual peculiarities	Important habitual characteristics	New characteristics
Patient 1	Uncertain mentalization	Diffuse anxieties	Hysterical character trait	Reduction of the manifestations of hysterical traits
Patient 2	Poor mentalization	Defensive denial mechanism	Diffuse anxieties (loss of object)	Elaboration of mourning
Patient 3	Uncertain mentalization	Depressive status	Object anxieties	Reduction in symptoms of depression
Patient 4	Poor mentalization	Hypochondriac	Diffuse anxieties	Decrease in progressive disorganization
Patient 5	Uncertain mentalization	Denial of reality (not pathological)	Obsessive character trait	Knowledge of the set of mental functioning

## Discussion

This current pilot study aimed to evaluate the effect of 1 year of regular and weekly psychotherapy sessions in patients with refractory vasovagal syncope, diagnosed by clinical history and positive tilt table test, in comparison to the previous year and to a control group, not submitted to psychotherapy. Most patients with VVS respond to previously proposed general measures and/or pharmacotherapy, targeting the improvement of blood volume distribution, but some of them are resistant, probable because of other triggering factors, in addition to central orthostatic hypovolemia. Although the same medical care, recommended in the international guidelines, was applied in both groups, there was no statistical difference in the recurrence of events in the control group during the follow-up, but there was a significant reduction of more than 50% in the occurrence of syncope and near-syncope in the intervention group. This reduction was associated with a significant improvement in quality of life, assessed by SF-36 questionnaire, while in the control group no statistical difference in QoL was observed. Both co-primary endpoints were better among the patients that underwent psychotherapy compared to the control group. The results were statistically significant even considering a Bonferroni correction to the tests of the primary endpoints.

Vasovagal syncope is a symptom that can be related to a wide spectrum of triggers and individual conditions, such as genetic predisposition to impaired peripheral vasoconstriction, dehydration, even anatomical damage to the autonomic ganglia caused by infectious agents. The autonomic nervous system (ANS) is a complex network and has reciprocal connections throughout the body, including the limbic system, which is the centre of human emotions, social behaviours, and unconscious survival responses. This association between better quality of life and lower recurrence of syncope observed in the study, reinforces the connection between emotional status and cardiovascular response. The interactions between human brain and cardiovascular system are well-known and may elicit diverse clinical manifestations. Cerebral areas related to emotions can affect the heart rate and blood pressure, throughout the autonomic nervous system. Experimental studies have shown that the stimulation of some sites, such as the insular cortex can potentially trigger fatal cardiac arrhythmias^15^.

Epidemiological studies demonstrated that severe stressed conditions can induce cardiac failure, such as Takotsubo Disease, also called Stress Cardiomyopathy, which aetiology is a global ischemia due to diffuse vasoconstriction of cardiac small arteries^16^. Endogenous Depression is also a psychological condition related to higher cardiovascular mortality among patients with or without previous cardiovascular diseases^17^. Of note, the central nucleus of the autonomic nervous system is involved in a complex network, which has reciprocal connections with the cerebral cortex, the limbic system and the cardiovascular system, mediated by catecholamines. These interactions explain the clinical association between psychic disorders and the trigger of vasovagal syncope. In this type of syncope, frequent recurrences lead to poor quality of life and impairment of the emotional status, which may contribute to new events and, consequently, to the development of a vicious cycle^4–8^.

In the current pilot study, two main questions were assessed: if weekly sessions of psychotherapy could reduce recurrences of syncope and also improve quality of life. Since these two endpoints are supposed to be strongly correlated, an intervention that reduces recurrences would impact the quality of life and vice-versa. This relationship between recurrence and quality of life led to the decision of designing a study with both as co-primary endpoints. Although vasovagal syncope is a benign condition in terms of mortality, recurrence of events is highly relevant in increasing morbidity (such as physical trauma due to falls and accidents) and declining self-confidence, what seriously compromises patients’ autonomy and QoL. The consequences of syncope recurrences are directly related to the number of events and the consequent limitations caused. The restrictions were reported to be comparable to chronic systemic diseases, such as arthritis, endogenous depression and severe renal failure^9–11^. It is also important to remind that the impairment on quality of life occurs even in the inter-crisis period, once the patient feels constantly concerned to perform daily life activities.

In this trial, all patients in both groups had a confirmed diagnosis of vasovagal syncope, received the same educational intervention and 40% of the patients were medicated with fludrocortisone. Thus, the patients selected to participate in this study, despite the introduction of conventional standard therapy and the medical follow-up in a reference service by a specialized team, remained very symptomatic. This was the reason why they were considered a valuable sample to evaluate the potential impact of psychotherapy on the study outcomes: recurrence of events (syncope and near syncope) and in their QoL. During the sessions, the patients could share their limitations and fears, trying to minimize emotional triggers and improve self-confidence. All the patients had significant traumatic events before the first syncope episodes and the psychotherapy modified their mental structure assessed by the Pierre Marty classification^12^. As a consequence of an improvement in mental health status, the intervention group had less recurrences and a better quality of life during the follow-up. This promising alternative of adjunct therapy is relevant for clinical practice in refractory cases of VVS, since the current options for this population, including pharmacologic treatment, have limited benefit^4–6,18–20^.

### Limitations

The small number of patients included in this pilot randomised trial is a limitation to its external validation. Nevertheless, there is a gap in the literature regarding the specific effect of psychotherapy on the set of treatment of patients with recurrent vasovagal syncope and this study brings also a unique information of an intervention, based on psychoanalysis, which is not commonly assessed in randomised trials. The results could be interpreted as a “proof of concept” of benefit of this type of psychotherapy in recurrent vasovagal syncope. However, the open-label methodology without a “sham” procedure in the control group restrain this affirmation. In addition, during the follow-up, was found that all patients had significant psychological traumatic events temporally related to the first syncope episodes. Thus, the results of the current study could not be applicable to the overall refractory VVS population, but specifically to a group of patients with psychological traumatic events which were recognized during the sessions of psychotherapy. Finally, beyond the patient selection, the intervention was particularly specific, since it was performed by a same and experienced psychologist. Thus, it should be further studied for additional validation in a more heterogeneous group of patients, using different approaches in psychotherapy during a longer follow-up period. Nevertheless, in patients with VVS similar to the study population, who already benefit from psychotherapy to manage psychological traumas, weekly sessions of psychotherapy could be considered an attractive adjunct strategy to improve quality of life and reduce syncopal events.

## Conclusion

In this randomised, pilot, open-label, controlled trial, patients with recurrent refractory vasovagal syncope that underwent regular psychotherapeutic intervention had significant reduction in the recurrence of events and an improvement on quality of life during 1 year of follow-up.
